# MAD-Onto: an ontology design for mobile app development

**DOI:** 10.3389/frai.2025.1508225

**Published:** 2025-02-03

**Authors:** Bilal Abu-Salih, Marwan Al-Tawil, Ansar Khoury, Dana A. Al-Qudah, Isra’a Abu Zaid, Marwa Alabdale, Dima Azar

**Affiliations:** King Abdullah II School of Information Technology, The University of Jordan, Amman, Jordan

**Keywords:** mobile app development, ontology design, knowledge representation, SWRL rules, emerging technologies

## Abstract

**Introduction:**

Mobile app development has rapidly evolved into a crucial aspect of modern technology, driving innovation across various industries and transforming user experiences globally. The dynamic nature of mobile technology requires developers to navigate a complex landscape of platforms, devices, and user requirements. Effective management and sharing of knowledge are essential to address these challenges, ensuring streamlined development processes and enhanced collaboration among stakeholders.

**Methods:**

To this end, ontologies have emerged as powerful tools for structuring and standardizing domain-specific knowledge. This paper introduces MAD-onto, a comprehensive ontology designed specifically for the mobile app development domain. The ontology is constructed by identifying key concepts, defining classes and their hierarchies, establishing class properties, and creating instances relevant to mobile app development. To ensure robustness, the ontology is evaluated using a multi-criteria evaluation metric, focusing on consistency, completeness, conciseness, expandability, and sensitiveness. Additionally, SWRL rules are applied to validate and enforce logical constraints within the ontology.

**Results:**

Through these rigorous evaluation methods, MAD-onto demonstrates its utility in providing a structured framework for the mobile app development lifecycle, facilitating better decision-making, collaboration, and efficiency.

**Discussion:**

The findings highlight the significance of ontology-driven approaches in addressing the complexities of mobile app development and set a foundation for future research and advancements in this field.

## 1 Introduction

The rapid evolution of mobile technologies has led to an exponential increase in the development and usage of mobile application (Pahlavan and Krishnamurthy, 2021). In this highly competitive environment, developers constantly seek innovative solutions to enhance app functionality, user engagement, and overall performance (Zhang et al., 2023; Hsu, 2023; Khan et al., 2023). However, the vast array of tools, frameworks, and best practices available presents a significant challenge in identifying the most suitable options for specific development needs (Yahya et al., 2023; Goh et al., 2023; Nazir et al., 2024; Sidiq et al., 2024). Addressing this challenge requires a comprehensive, structured approach to managing and utilizing the extensive knowledge base of mobile app development. To navigate this complex environment, developers should consider certain structured approaches including the following aspects: (1) Before starting the development process, it is crucial to understand the target audience, their needs, and the competitive landscape (Asamoah et al., 2024). (2) Developers have to decide whether to build a native, hybrid, or web-based application, each with its own set of advantages and trade-offs (Carbajal, 2024). (3) Developers have to select appropriate tools and frameworks such as iOS, Android, React Native, Unity, Xamarin, and Cordova, which cater to different development needs. (4) Developers have to implement best practices such as strategic planning, competitor analysis, prioritizing design, creating mockups and prototypes, choosing the right technology, and conducting multiple tests (Larrea et al., 2024). (5) Developers have to stay updated with trends and emerging technologies, and keep abreast of the latest trends and insights in mobile app development frameworks to ensure the use of modern and efficient technologies.

Ontologies have emerged as a powerful tool for organizing and structuring domain-specific knowledge, enabling more efficient retrieval and application of information (Abu-Salih, 2022; Abu-Salih and Alotaibi, 2023; Al-Hassan et al., 2023; Abu-Salih et al., 2023). In the context of mobile app development, an ontology can serve as a knowledge-based framework to support personalized recommendations. For instance, by understanding the user’s preferences and behavior within the app, the ontology can provide recommendations tailored to the user’s specific needs and interests. Ontologies can also enhance semantic search capabilities. By mapping these behaviors onto the ontology, the app can provide more relevant search results, improving the user experience. Moreover, ontologies can improve the overall development process. Developers can use the ontology to understand the target audience, their needs, and the competitive landscape. They can also decide whether to build a native, hybrid, or web-based application, each with its own set of advantages and trade-offs. Developers can select appropriate tools and frameworks such as iOS, Android, React Native, Unity, Xamarin, and Cordova, which cater to different development needs. They can implement best practices such as strategic planning, competitor analysis, prioritizing design, creating mockups and prototypes, choosing the right technology, and conducting multiple tests. Developers can stay updated with trends and emerging technologies, and keep abreast of the latest trends and insights in mobile app development frameworks to ensure the use of modern and efficient technologies.

This paper presents MAD-Onto, a domain-specific ontology designed to encapsulate the diverse and complex knowledge associated with mobile app development. MAD-onto aims to standardize and formalize the terminology, concepts, and relationships pertinent to this domain, thereby providing a robust foundation for developing intelligent systems and applications. The evaluation of MAD-onto encompasses several dimensions, including consistency, completeness, conciseness, expandability, and sensitivity. Consistency checks involve the use of automated reasoning tools such as FaCT++, HermiT, Pellet, RacerPro, and TrOWL to detect any logical contradictions within the ontology. Completeness is assessed by comparing the ontology against a set of predefined requirements or benchmarks established by domain experts, ensuring that all necessary concepts and relationships are captured. Conciseness is verified by ensuring that the ontology includes only essential concepts and relationships, avoiding redundancy and facilitating ease of use. Expandability is evaluated by examining the ontology’s ability to accommodate new concepts and relationships without significant restructuring, thus ensuring its adaptability to evolving domain knowledge. Sensitivity analysis is conducted to determine how changes to the ontology impact its core structure, ensuring robustness and flexibility. Additionally, various quantitative metrics such as Vocabulary Size (VS), Connectivity Ratio (CR), Tree Impurity (TIP), and Entropy of Ontology Graph (EOG) are employed to provide a comprehensive evaluation of the ontology’s structure and complexity. This multi-faceted evaluation approach ensures that MAD-onto is not only theoretically sound but also practically useful, reliable, and adaptable to the dynamic nature of mobile app development. The evaluation of MAD-onto also incorporates the use of SWRL (Semantic Web Rule Language) rules to further ensure the ontology’s robustness and correctness. SWRL rules allow for the expression of complex logic that goes beyond the capabilities of OWL (Web Ontology Language), providing a means to enforce constraints and verify logical consistency within the ontology. The key contributions of this paper are summarised as follows:

- MAD-onto offers a domain-specific ontology that captures the complexities of mobile app development, including emerging technologies such as augmented reality, artificial intelligence, and blockchain.
- Unlike previous ontologies, MAD-onto integrates SWRL rules to enforce logical constraints, such as task sequencing, resource allocation, and skill matching.
- Mad-onto is evaluated using a robust set of metrics, including VS, TIP, CR, and EOG. These metrics ensure the ontology’s consistency, completeness, and adaptability.

The remainder of this paper is organized as follows: Section 2 provides a detailed overview of the related work in the field of mobile app development and ontologies. Section 3 shows the overall methodology used in this research. Section 4 describes the design and implementation of MAD-onto, including its structure, key concepts, and evaluation metrics. Section 5 illustrates the evaluation metrics used to measure the applicability of MAD-onto. Finally, Section 6 concludes the paper with a discussion of the implications, limitations, and potential future directions of this research.

## 2 Related works

Ontologies have emerged as a crucial tool in the field of mobile app development, providing a structured framework for knowledge representation and sharing. The use of ontologies in this domain facilitates enhanced communication, interoperability, and knowledge management among various stakeholders. Several studies have underscored the importance of ontologies in improving the efficiency and effectiveness of the mobile app development process (Iqbal et al., 2021; Iqbal et al., 2022; Braham et al., 2022; OS, 2021; Huitzil et al., 2020; de Freitas et al., 2023). For example, a recent study by Braham et al. (2022) examined the role of design patterns and ontology models in the generation of mobile applications. The study introduced an ontology-based framework to represent, design, and support the adaptation of user interfaces in mobile applications by using design patterns according to user needs or preferences and the context around them. This work underscores the importance of ontologies in creating adaptive mobile applications that can respond to various user needs and different context scenarios. In the realm of e-learning, a study discussed the use of ontologies for developing a mobile application of the corporate e-learning system in the process of adaptation of companies’ new employees (Bakanova et al., 2019). The study highlighted the role of ontologies in building individual learning paths in the process of employee adaptation. This demonstrates the versatility of ontologies in various domains, including corporate training and employee adaptation. Another study by Johannsen et al. (2023) proposed a generalizable success model for mobile apps with a focus on first-year students. The study analyzed those factors that influence student satisfaction with such an app, the intention to reuse the app, and—foremost—students’ learning effectiveness. Although not directly related to ontologies, this study provides valuable insights into the factors that contribute to the success of a mobile app, which can be useful in the development of ontology-based mobile apps.

In fact, this paper is a report of work in progress the aim of which is develop a new recommender system framework that is designed to provide mobile app developers with a distinctive platform to browse and search for personalised artifacts (Abu-Salih et al., 2021). The proposed system makes use of ontology and semantic web technology as well as machine learning techniques. The new recommender system framework comprises the following components: (i) Domain knowledge inference module: including various semantic web technologies and lightweight ontologies. (2) Profiling and preferencing: a new proposed time-aware multidimensional user modelling. (3) Query expansion: to improve and enhance the retrieved results by semantically augmenting users’ query. (4) Recommendation and information filtration: to make use of the aforementioned components to provide personalised services to the designated users and to answer a user’s query with the minimum mismatches. This study underscored the potential of ontologies in enhancing the efficiency and effectiveness of the mobile app development process, particularly in the context of personalised recommender systems. It also highlights the importance of semantic web technologies and machine learning techniques in this domain. In conclusion, the use of ontologies in mobile app development has been recognized as a valuable tool for enhancing communication, interoperability, and knowledge management. Recent studies have demonstrated the versatility of ontologies in various domains, including adaptive mobile applications, corporate training, and employee adaptation. However, more research is needed to fully explore the potential of ontologies in mobile app development.

### 2.1 A comparison with existing ontologies

Unlike existing ontologies such as Pani and Mishra (2016), which focuses narrowly on native app development workflows, MAD-onto is domain-agnostic and extends to emerging technologies like AI, AR, and blockchain. This broader scope accommodates the dynamic and evolving nature of mobile app development, ensuring relevance across diverse use cases. Further, MAD-onto employs a multifaceted evaluation strategy encompassing structural (Tree Impurity, Connectivity Ratio), semantic (SWRL rules), and usability (Expandability) metrics. By contrast, prior ontologies often rely on *ad hoc* or superficial evaluations, limiting their robustness for practical deployment. Finally, MAD-onto ontology builds on existing works (Norki et al., 2020), offering a more detailed and comprehensive representation of the mobile app development domain. It encompasses various aspects such as development approaches, developer expertise, testing strategies, and critical features. By integrating SWRL rules, MAD-onto ensures the consistency, completeness, and reliability of the ontology, making it a valuable tool for researchers and practitioners in the field. The MAD-onto ontology also addresses the gaps identified in previous works by providing a more granular and fine-grained representation of the domain. It includes detailed classes and properties, organized hierarchically to facilitate ease of use and understanding. The use of automated reasoning tools for consistency checking further enhances the ontology’s robustness, ensuring that it meets the intended requirements and constraints. These capabilities are absent in most prior works, which rely solely on taxonomical or descriptive models.

## 3 Methodology

This study employs the Design Science Research Methodology (DSRM) approach, which has been established as a standard research paradigm in the Information Systems (IS) field to provide researchers with a structured framework for creating constructs, models, methods, and instantiations (von Alan et al., 2004). Design Theory, as presented by Jones and Gregor (2007) and Venable (2013), complements this methodology by detailing the essential components for articulating a design theory, while also simplifying its formulation and addressing key issues. Peffers et al. (2007) argue that DSRM is particularly effective for designing and evaluating artifacts that address real-world problems, offering a systematic framework that ensures both rigor and practical relevance.

**Figure 1:**
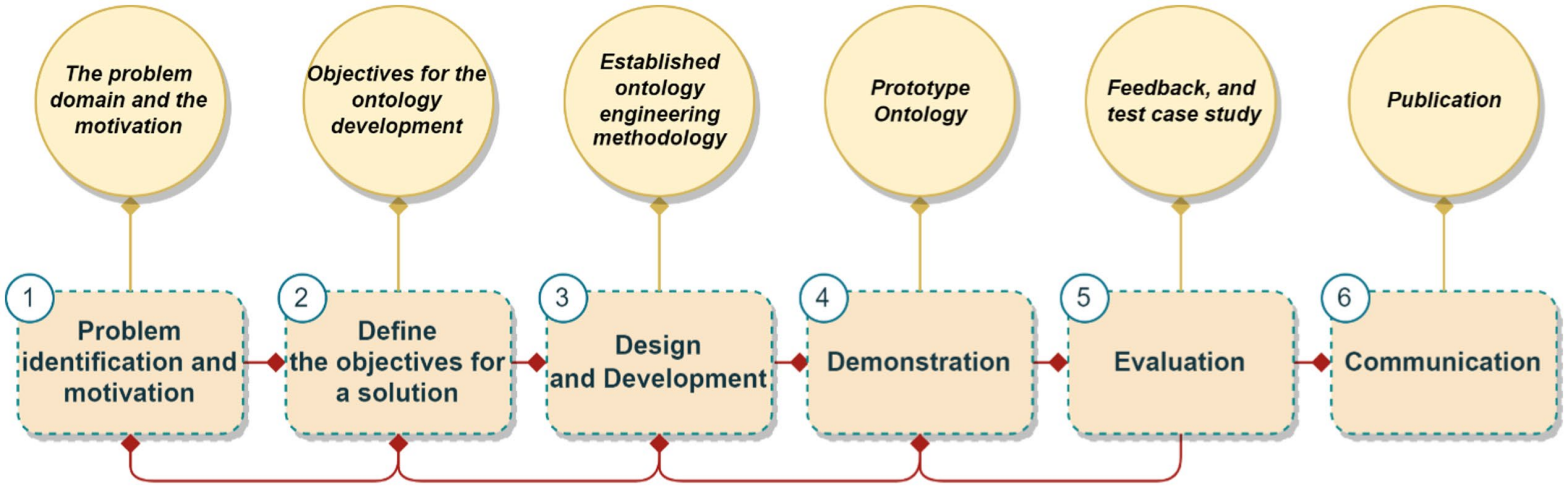
DSRM methodology [prepared by the authors based on the steps discussed in Peffers et al. (2007)].

As depicted in Figure 1, DSRM is iterative, leveraging feedback from each activity to inform and refine subsequent steps. The ultimate aim is to develop a design theory that can be generalized and applied to similar problems, and to create an artifact that practically and effectively addresses the identified issue. The proposed methodology includes the following six main activities:

1. Problem Identification and Motivation: This involves identifying and defining the problem that the research aims to solve, which sets the stage for the entire research process. Relevant activities include:

- Conducting a comprehensive literature review to identify gaps in existing ontologies related to mobile app development.
- Highlighting deficiencies in current ontologies, such as their limited scope in conceptualizing specific roles, tools, and development processes in mobile app development.

1. Define the Objectives for a Solution: Formulating clear and concise objectives for the proposed solution based on the problem identified. Relevant activities include:

- Developing a proof-of-concept ontology that integrates key entities and concepts relevant to mobile app development, such as development approaches, tools, and developer roles.
- Identifying key concepts and relationships within the domain, such as different development methodologies and the tools used by developers.

1. Design and Development: Creating an artifact or solution to address the identified problem using existing theories, frameworks, and best practices. Relevant activities include:

- Designing and developing the ontology using established methodologies such as the NeOn Methodology.
- Creating an ontology (MAD-Onto) with elements such as concepts (classes), attributes (properties), restrictions (facets), and instances (individuals).
- Utilizing software tools like Protégé to construct the ontology.

1. Demonstration: Showcasing and evaluating the developed artifact to stakeholders and experts to demonstrate its effectiveness. Relevant activities include:

- Developing a prototype ontology as a proof-of-concept to illustrate its capability in enhancing mobile app development processes.
- Applying the ontology to real-world scenarios, such as recommending development tools and frameworks based on developer profiles and project requirements.

1. Evaluation: Determining whether the developed artifact meets the objectives and provides a satisfactory solution. The evaluation process can include user studies, controlled experiments, or case studies. Relevant activities include:

- Conducting empirical studies and surveys to evaluate the ontology’s effectiveness and its impact on the mobile app development domain.
- Integrating various evaluation metrics to assess the ontology’s performance.

1. Communication: Disseminating the research findings and insights gained from the design and development of the artifact to the wider community. Relevant activities include:

- Publishing manuscripts and articles that discuss the need for, design approach of, and usefulness of the developed ontology.
- Facilitating the exchange of information and knowledge among academics, practitioners, and researchers.

## 4 MAD-Onto: an ontology design for the mobile app development

Several methodologies can be adopted to design and construct domain ontologies, including: (i) Top-down methodology: Starts with a high-level conceptual framework, such as a philosophical theory or a domain-specific taxonomy, and refines it through iterative feedback from domain experts, adding more detailed concepts and relationships; (ii) Bottom-up methodology: Begins with a large collection of individual concepts and facts, such as those extracted from natural language texts or existing databases, and clusters them into more abstract categories based on their similarities and differences and (iii) Mixed methodology: Combines top-down and bottom-up methodologies in the ontology design.

This study integrates a top-down methodology, namely METHONTOLOGY (Fernández-López et al., 1997), with a mixed-based methodology, Cyc 101 (Lenat and Guha, 1993), to construct MAD-Onto. The process of constructing MAD-Onto is divided into four main steps:

1. Identifying the topic and extent of the ontology: This involves defining the scope of the ontology, including the concepts and relationships that will be included.
2. Ontology reuse: Existing ontologies that are relevant to the domain are identified and integrated into the new ontology to avoid duplicating effort.
3. Conceptual model creation: The concepts and relationships identified in the first two steps are used to create a conceptual model of the ontology.
4. Ontology evaluation: The ontology is evaluated to ensure that it accurately represents the domain of interest and meets the needs of its intended users.

The “Protégé” tool is used to build the ontology. Protégé is a free, open-source ontology editor and a knowledge management system that allows interaction with other reasoning tools and incorporates business principles for inference. It supports the most current WWW Consortium RDF and OWL 2 Web Ontology Language standards, ensuring that the ontology is compatible with current web technologies. The following subsections will detail the actions taken to build MAD-Onto, providing a step-by-step account of how the ontology was constructed using the METHONTOLOGY and Cyc 101 methodologies and the Protégé tool. This includes specific decisions made during the process, challenges encountered and how they were overcome, and any adaptations made to the methodologies to suit the specific needs of the MAD domain. The final ontology would then be evaluated to ensure it meets the requirements defined at the start of the process.

### 4.1 Identifying the domain and extent of the ontology

This stage specifies the domain that the ontology will conceptualize as well as the queries that the designed ontology will address. Table 1 demonstrates our response to the queries used to identify the ontology’s domain and scope.

**Table 1:** Key questions for identifying the domain and scope of the ontology.

Query	Response
What is the domain that the ontology will cover?	This study aims to cover the specific domain of Mobile App Development by designing a specialized ontology, referred to as MAD-Onto.
What is the purpose and goal of this ontology?	The primary goal of MAD-Onto is to provide a comprehensive understanding of Mobile App Development, thereby facilitating more effective and efficient app development processes.
Who are the intended users of this ontology?	MAD-Onto is designed to benefit both academic researchers and industry practitioners who are interested in developing a conceptual model for Mobile App Development. Given the rapid advancements in this field, the proposed ontology is designed to be adaptable and expandable, allowing for the incorporation of new concepts, properties, and examples in the future.
What key questions can the integrated knowledge in the ontology answer?	What are the core concepts that define and conceptualize domain-specific Mobile App Development? How can the proposed ontology be utilized to enhance the development and deployment of domain-specific mobile apps? How can the ontology be leveraged to model the interaction and behavior of users within mobile apps? How can the ontology contribute to the standardization of Mobile App Development processes? How can the ontology support the integration of emerging technologies in Mobile App Development?

### 4.2 Ontology reuse

Ontology reuse is a critical aspect of ontology development that aims to leverage existing ontologies to reduce the effort needed to model a new domain and increase interoperability across applications (Carriero et al., 2020). It involves building a new ontology by maximizing the adoption of pre-used ontologies or ontology components (Halper et al., 2023). This not only reduces the human labor involved in formalizing ontologies from scratch but also increases the quality of new ontologies because the reused components have already been tested. In the context of Mobile App Development, several attempts have been made to develop ontologies that capture and conceptualize this domain (Pardo et al., 2023). These efforts have primarily focused on defining interaction patterns, user behaviors, and technical specifications of mobile apps (Braham et al., 2022; Werth et al., 2019; Norki et al., 2020; Iqbal et al., 2022). For instance, some studies have explored the role of design patterns and ontology models in generating mobile applications that can adapt at runtime to various user needs, different context scenarios, interactive design modes, or technology requirements (Braham et al., 2022).

However, despite these important efforts, there has been a gap in the development of an ontology for domain-specific MAD that is dedicated to standardizing and formalizing the specified domain knowledge. Such an ontology provides a structured and standardized representation of the domain, facilitating more effective and efficient app development processes. This study aims to address this gap by benefiting from other seminal works to develop a fine-grained ontology that conceptualizes domain-specific MAD. The goal is to create an ontology that not only captures the key concepts and relationships in this domain but also provides a standardized and formalized representation of this knowledge. Also, the proposed ontology combines top-down (METHONTOLOGY) and mixed-based (Cyc 101) methodologies, ensuring a comprehensive and balanced approach to ontology construction. This would enable developers to better understand the domain, make more informed decisions during the development process, and ultimately create more effective and user-centric mobile apps.

### 4.3 Development of a conceptual model

Designing a conceptual model for MAD-Onto comprises the following actions:

- Enumerate Key Terms in the Ontology: Identify and list critical terms or concepts relevant to mobile app development. These terms should cover various aspects such as development processes, app features, user interactions, and technical requirements. This foundational vocabulary forms the basis of the ontology, enabling accurate representation and communication of concepts within the domain. For MAD-Onto, this includes: (1) Development Phases: Requirements analysis, design, implementation, testing, deployment, maintenance. (2) App Features: User interface components, backend services, security features, performance metrics. (3) User Interactions: User actions, feedback mechanisms, usability metrics. (4) Technical Requirements: Programming languages, frameworks, platforms, databases.
- Define Classes and Their Hierarchy: identify the key concepts and entities that are relevant to the domain and organize them into a hierarchical structure. Defining classes and their hierarchy is a critical step in designing the MAD-Onto ontology. This involves identifying key concepts and entities relevant to mobile app development and organizing them into a structured hierarchy. The hierarchical structure begins with broad, general classes and progressively narrows down to more specific subclasses, ensuring a clear and logical organization of domain knowledge. By defining classes and their hierarchy in this structured manner, MAD-Onto can effectively capture the complexities of mobile app development. This hierarchical organization not only aids in understanding the relationships between different concepts but also facilitates the integration and retrieval of knowledge within the domain.
- Define Class Properties—Slots: Define the attributes and relationships of the classes, known as properties or slots. These can be object properties (relationships between classes) and datatype properties (attributes of a class). Examples include: develops (connects a developer to a mobile application), usesFramework (connects a mobile application to a frontend framework). Examples of datatype properties: appName (the name of the mobile application, type: string), releaseDate (the release date of the application, type: date), and version (the version number of the application, type: string).
- Define the Facets of Slots: Define the type and value constraints for each property. This ensures that the data within the ontology is structured and validated. Examples include MessageContent: A property with a type of “string,” capturing textual content, and ReleaseDate: A property with a type of “date,” indicating when the mobile application was released.
- Create Instances: Populate the ontology with individual instances representing real-world entities relevant to mobile app development. These instances can be created manually or through automated processes such as data extraction or machine learning algorithms. Examples of instances include: An instance of MobileApplication representing a specific app like “MyFitnessApp.” An instance of FrontendFramework representing “ReactNative.” An instance of Developer representing a specific developer or development team.

Our MAD-Onto is a structured representation of the Mobile App Development domain, incorporating concepts, relationships, attributes, and examples. The development process involved a meticulous investigation of academic literature and corporate reports to extract the necessary technical terminology. The process began with a thorough review of existing academic papers and corporate reports. This step was crucial to gather a comprehensive set of technical terms and concepts that are pivotal in the Mobile App Development field. Protégé, an open-source platform, was employed for its user-friendly interface, which facilitates the creation, editing, and visualization of ontologies. This tool supports the most current standards, such as RDF and OWL 2 Web Ontology Language, making it a suitable choice for developing MAD-Onto. The ontology was described and modeled using OWL-DL to ensure that it is well-defined, consistent, and capable of being shared and reused. OWL-DL provides the expressiveness needed to capture the complexity of the domain while ensuring computational completeness and decidability. By following these steps, MAD-Onto was constructed to serve as a reliable and standardized knowledge base that can enhance the understanding and efficiency of mobile app development processes. It stands as a testament to the rigorous methodology applied, combining theoretical research with practical application to produce a valuable resource for both academia and industry. The following is a description of each of the high-level classes in our MAD-Onto ontology:

- EmergingTechnology: This class encompasses the cutting-edge technologies that are currently shaping the future of digital transformation. It includes Artificial Intelligence with its sub-classes like Computer Vision, Machine Learning, and Natural Language Processing which are revolutionizing how machines interpret and interact with the world. Augmented Reality, Blockchain, Internet of Things, and Virtual Reality are also part of this class, each representing a significant leap forward in their respective fields.
- MobileApp: The MobileApp class represents the software applications designed to run on mobile devices. It covers the entire lifecycle of a mobile app, from conception to deployment, and includes various attributes such as platform, functionality, and user interface.
- Person: Under the Person class, there are two main subclasses: MobileAppTester, with roles like QAEngineer, and MobileAppDeveloper, which includes DevOpsEngineer, FullStackDeveloper, BackendDeveloper, and FrontendDeveloper with a further subclass of UIUXDesigner. This class encapsulates the human resources involved in mobile app development and testing.
- SupportingTechnology: This class includes technologies that support mobile app development, such as API_Development, CloudService, and Database technologies, with subclasses like NoSQL and RelationalDB. These technologies provide the necessary infrastructure and services for mobile apps to function and scale.
- AppMarket: The AppMarket class represents the digital distribution platforms where mobile apps are made available for users. It includes various marketplaces like Google Play Store and Apple App Store, and covers aspects related to app submission, review, and distribution.
- DevelopmentApproach: This class outlines the methodologies and strategies used in mobile app development, such as CrossPlatform development with various techniques like JavaScriptBridging and Hybrid models, MobileWeb approaches including AdaptiveDesign and ResponsiveDesign, and Native development specific to platforms like Android and iOS.
- DevelopmentEnd: The DevelopmentEnd class is divided into BackEndDevelopment and FrontEndDevelopment, each covering the respective aspects of app development. FrontEndDevelopment includes sub-classes like FrontendArchitecture, UIUXDesign, FI-Framework_Library, FI-Language, and SDK.
- DevelopmentLanguage: This class categorizes the programming languages used in mobile app development into BackendLanguage and FrontendLanguage. Subclasses like CompiledLanguage, InterpretedLanguage, Markup, Scripting, and Stylesheet further specify the types of languages used.
- DevelopmentLibrary: The DevelopmentLibrary class includes libraries and frameworks that provide pre-written code and templates for app development. It’s divided into BackendFramework and FrontendFramework, with subclasses such as Component-based and Hybrid frameworks.
- DevelopmentToolCost: This class deals with the financial aspect of mobile app development tools, considering the cost of acquisition, licensing, and maintenance of the development environments and tools used.
- DeviceAccessManager: The DeviceAccessManager class includes the management of access to device features such as CameraAccess, HardwareIntegration, LocationServices, PermissionsManagement, SensorAccess, and StorageAccess.
- ExecutionEnvironment: This class represents the environments in which mobile apps are executed, including MobileOS, Containerization, and RuntimeEnvironment.
- IDE: The IDE (Integrated Development Environment) class encompasses the software suites that provide comprehensive facilities to programmers for software development, including code creation, editing, and debugging.
- MemoryManagement: The MemoryManagement class includes the techniques and tools used to manage memory in mobile app development, such as AutomaticReferenceCounting, GarbageCollection, ManualMemoryManagement, and MemoryLeakDetection.
- MobilePlatform: The MobilePlatform class represents the operating systems for mobile devices, including Android, WindowsPhone, and iOS, each providing a unique ecosystem for mobile app development and deployment.

Each of these classes plays a crucial role in the ontology, representing a distinct aspect of the mobile app development domain, and together they form a comprehensive framework for understanding and organizing the knowledge in this field. MAD-Onto provides a standardized vocabulary for the mobile app development domain. This facilitates clear communication among developers, project managers, and stakeholders, ensuring that everyone has a common understanding of the terms and concepts used. By defining a clear structure of classes and relationships, MAD-Onto enables better collaboration between different teams and individuals involved in the app development process. It helps in aligning the efforts of designers, developers, and testers towards a unified goal. The ontology allows for the reuse of knowledge across different projects and applications. This not only saves time and resources but also promotes consistency in the development practices within the industry.

**Figure 2:**
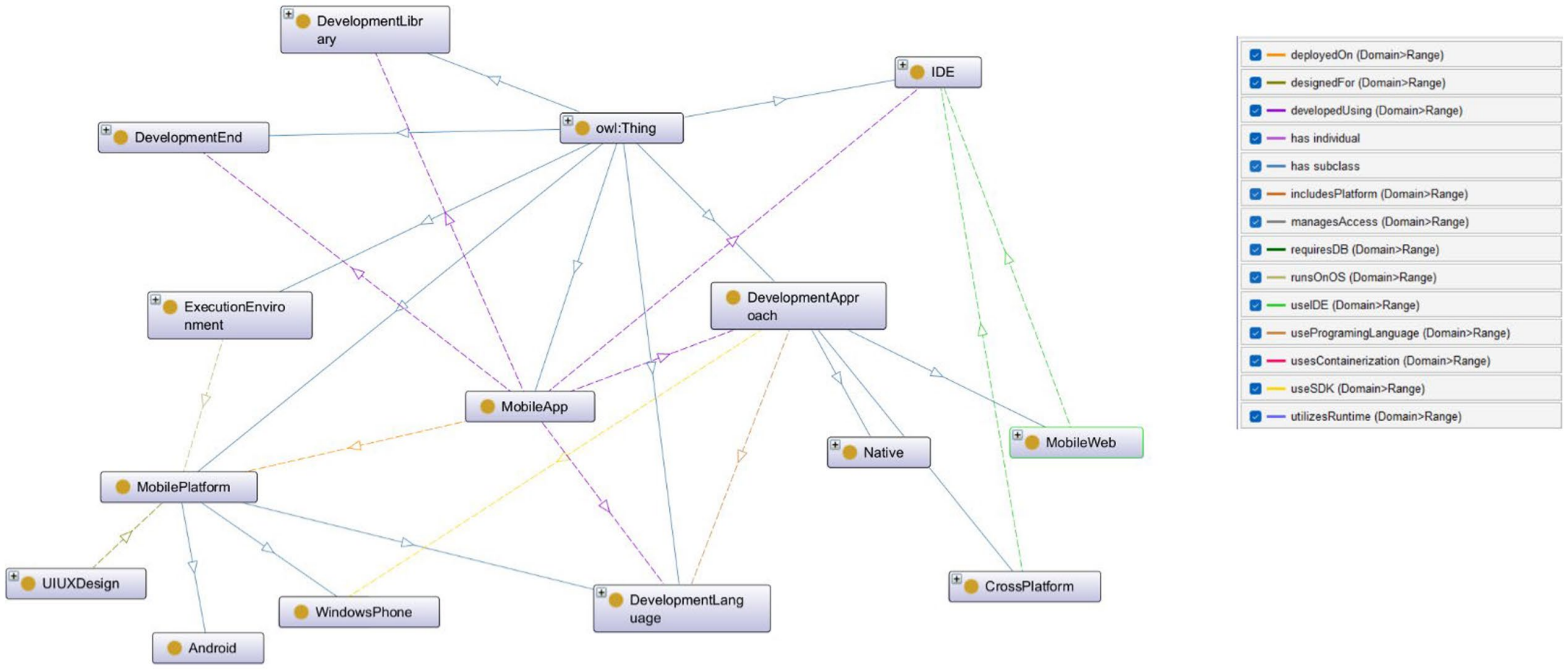
A snapshot of MobileApp class and its interconnected classes and subclasses in MAD-Onto.

**Figure 3:**
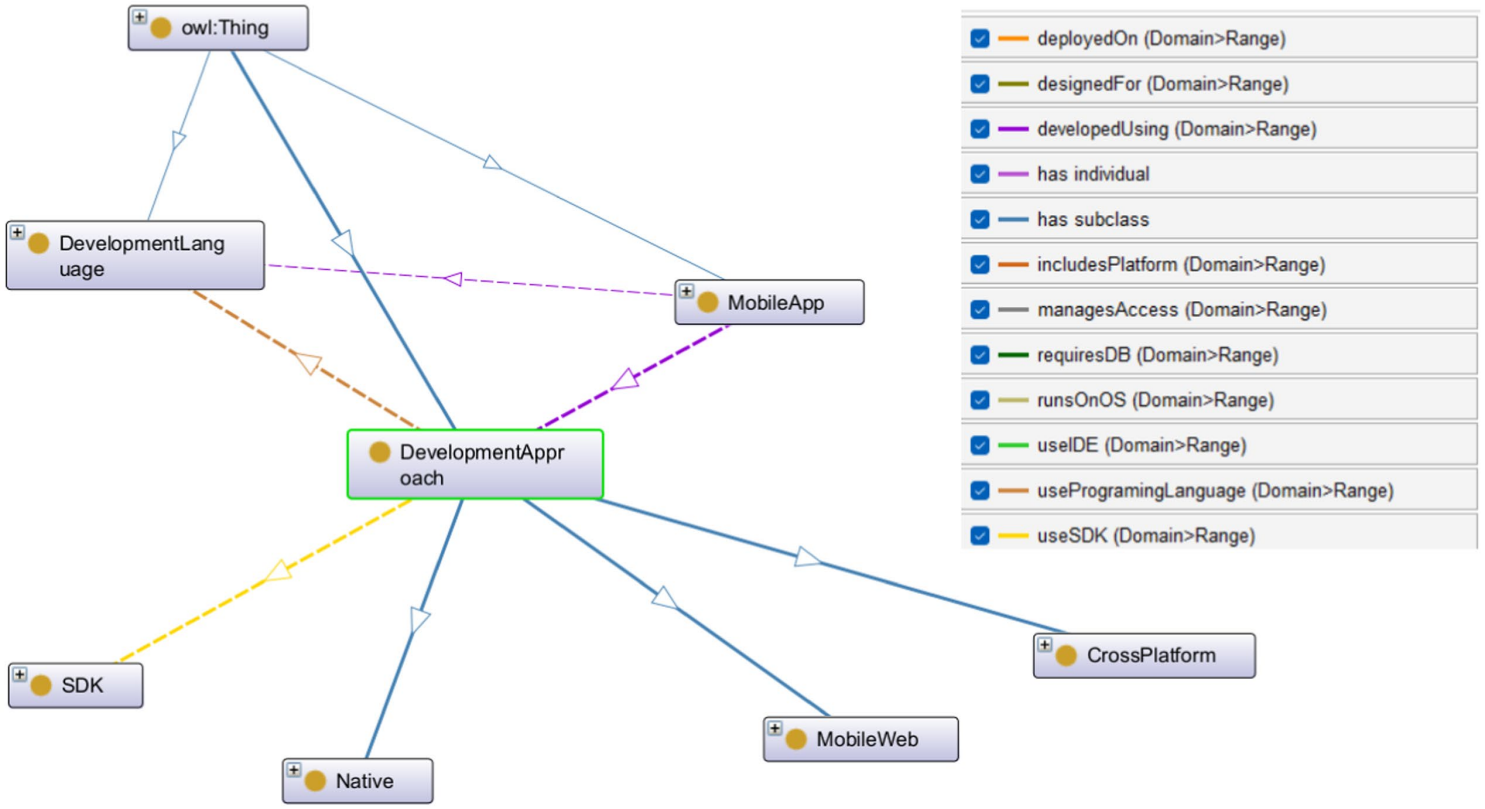
A snapshot of the DevelopmentApproach class and its interconnected classes and subclasses in MAD-Onto.

**Figure 4:**
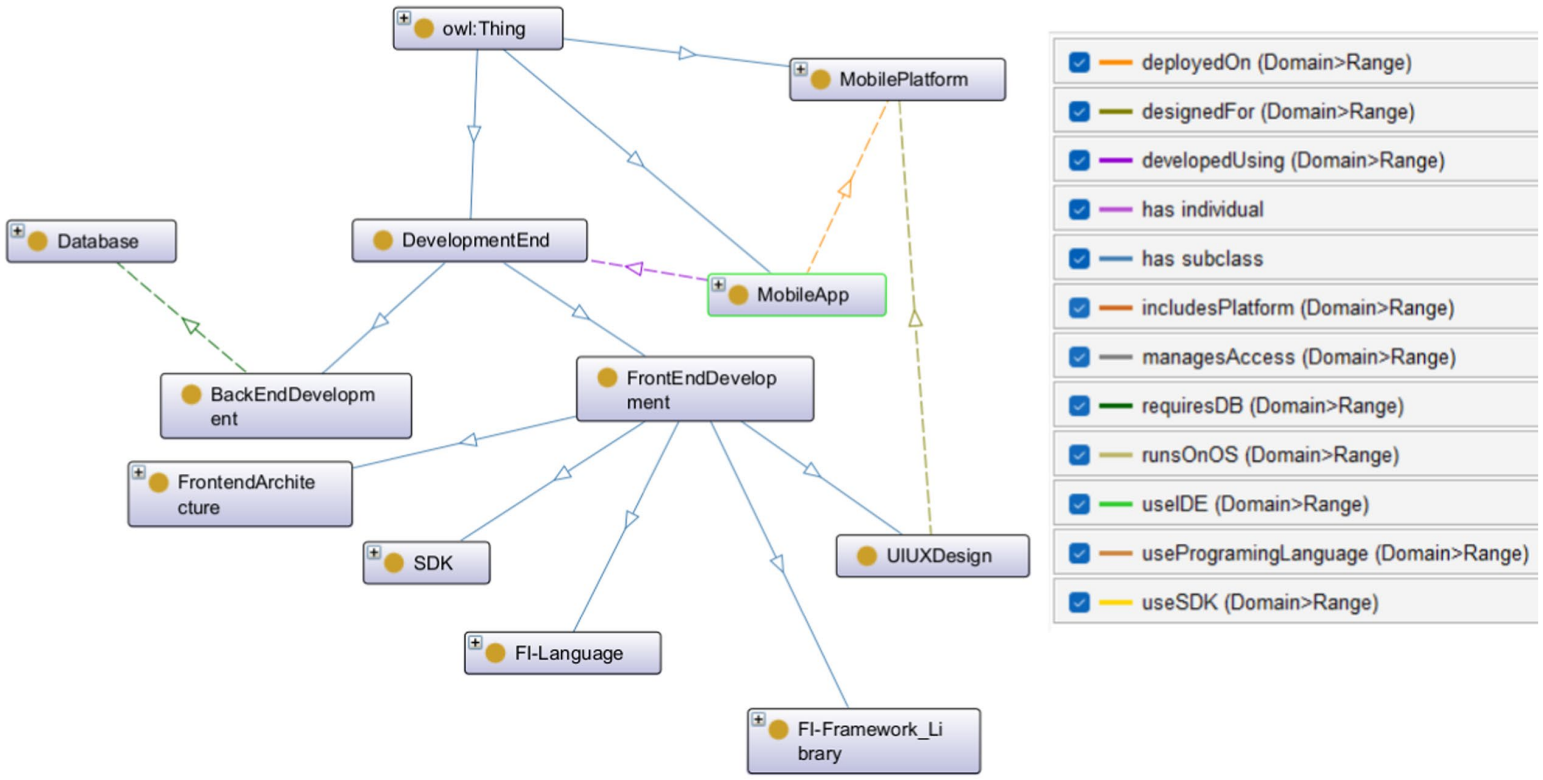
A snapshot of the DevelopmentEnd class and its interconnected classes and subclasses in MAD-Onto.

**Figure 5:**
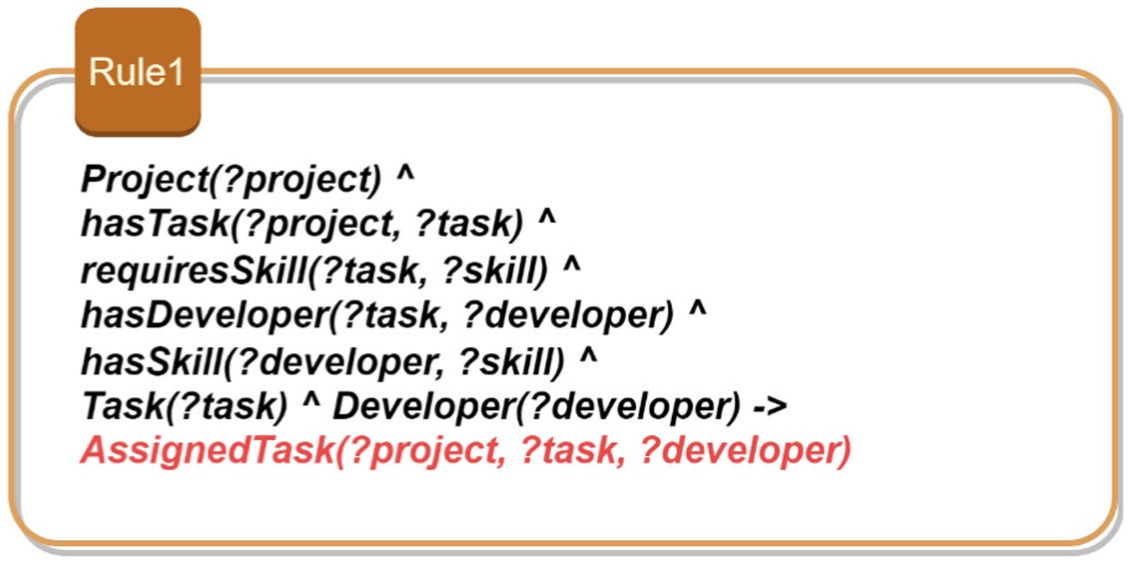
SWRL—Rule 1.

Figures 2–5 further illustrate the developed ontology including the key classes and the interrelated object properties. For example, Figure 1 shows MobileApp class which is the cornerstone of the MAD-Onto ontology, encapsulating all critical elements associated with mobile application development. This class serves as the primary hub, linking various components, processes, and tools essential for creating, deploying, and maintaining mobile applications. By defining MobileApp as the central entity, the ontology ensures a cohesive structure that integrates diverse aspects of mobile app development, offering a comprehensive view of the domain.

Figure 3 shows the DevelopmentApproach class and other several key attributes and relationships that illustrate its comprehensive role in mobile app development. The DevelopmentApproach class is intricately connected to other classes through various relationships:

- includesPlatform: This relationship links DevelopmentApproach to the MobilePlatform class, indicating the platforms that support different development approaches. This connection underscores the importance of platform compatibility and optimization in the selection of development methodologies.
- useProgramingLanguage: Through this relationship, DevelopmentApproach is connected to the DevelopmentLanguage class, specifying the programming languages associated with each development approach. This reflects the technical foundation and coding practices that underpin different development strategies.
- useSDK: By associating DevelopmentApproach with specific Software Development Kits (SDKs), this relationship highlights the tools and resources utilized within each approach, providing developers with pre-built components and functionalities tailored to their chosen methodology.
- developedUsing: This critical relationship associates DevelopmentApproach with DevelopmentLibrary, specifying the libraries and frameworks utilized during the development process. It reflects the technical infrastructure and resources that support each development approach.
- designedFor: This relationship connects DevelopmentApproach to the UIUXDesign class, emphasizing the importance of user interface and user experience considerations in different development strategies. It ensures that applications are not only functional but also user-friendly and aesthetically pleasing.
- runsOnOS: Linking DevelopmentApproach to the MobilePlatform class, this relationship identifies the operating systems (e.g., Android, iOS, Windows Phone) compatible with each approach. This connection highlights the importance of platform-specific optimization in mobile app development.

As depicted in Figure 4, the DevelopmentEnd class integrates various critical components of mobile app development, ensuring that the final product is robust, efficient, and user-friendly. This class plays a crucial role in the development lifecycle, as it brings together all the essential elements required to finalize and deploy a mobile application. The DevelopmentEnd class is characterized by several key attributes and relationships that detail its role and connections within the mobile app development ecosystem:

- useIDE: This relationship connects the DevelopmentEnd class to the IDE (Integrated Development Environment) class, highlighting the use of specific development environments essential for writing, debugging, and testing code. Examples of IDEs include Android Studio, Xcode, and Visual Studio, which provide developers with necessary tools and features to streamline the development process.
- useSDK: This relationship links the DevelopmentEnd class to the SDK (Software Development Kit) class, emphasizing the importance of SDKs in the development process. SDKs provide pre-built libraries, tools, and APIs that facilitate the development of mobile applications by offering functionalities such as push notifications, analytics, and authentication.
- developedUsing: This relationship associates the DevelopmentEnd class with the FI-Framework_Library and FI-Language classes, specifying the frameworks, libraries, and programming languages used during the final stages of development. Frameworks like React Native, Flutter, and libraries like Retrofit or Alamofire are examples that provide robust functionalities and simplify complex development tasks.
- requiresDB: This connection to the Database class underscores the necessity of database integration in the development end process. Databases are crucial for data storage, retrieval, and management, ensuring that the mobile application can efficiently handle user data, preferences, and other critical information.
- includesPlatform: This relationship indicates that the DevelopmentEnd phase includes considerations for the MobilePlatform class, ensuring that the application is compatible and optimized for specific mobile platforms such as Android, iOS, or cross-platform solutions.
- designedFor: This attribute links the DevelopmentEnd class to the UIUXDesign class, highlighting the significance of user interface and user experience design in the final stages of development. This ensures that the application not only functions well but also provides a seamless and intuitive user experience.

## 5 Evaluation

The evaluation of MAD-Onto is a critical phase in the Design Science Research Methodology, ensuring that the ontology not only meets its intended objectives but also contributes effectively to the domain of Mobile App Development. To assess the efficacy and impact of MAD-Onto, we employed a multi-faceted evaluation strategy, encompassing both qualitative and quantitative measures. There have been several reports in the literature on ontology evaluation measures (Yu et al., 2009; Alani and Brewster, 2006; d’Aquin et al., 2009; Dellschaft and Staab, 2008; Zavitsanos et al., 2010). Ontology evaluation establishes the quality of an ontology as well as whether its constraints and standards have been met. The following subsection discusses the evaluation metrics incorporated in this study.

### 5.1 Five criteria evaluation metric

This research employs an evaluation technique based on five criteria, adapted from Yu et al.’s (Yu et al., 2009) methodology:

1. Consistency: Consistency in an ontology refers to the absence of contradictions or conflicting information. A consistent ontology ensures that any logical inferences made using the ontology are reliable and accurate. Automated reasoning tools, such as FaCT++, HermiT, Pellet, RacerPro, and TrOWL, are employed to check that the axioms (statements) in the ontology do not lead to any logical contradictions. These reasoners examine the class, object, and data property structures, as well as class/object property claims and the presence of identical entities within the ontology. Ensuring consistency helps identify errors or gaps in the ontology’s design and implementation, allowing for refinement and improvement. For the MAD-Onto ontology, these reasoners confirm that there are no contradictory truths, ensuring the ontology is logically coherent.
2. Completeness: Completeness evaluates whether the ontology covers all the concepts and relationships relevant to its intended domain. This criterion assesses whether the ontology includes all the necessary knowledge required to support its intended tasks. The completeness of the MAD-Onto ontology is assessed by comparing it against a set of domain-specific requirements or benchmarks created based on expert knowledge. This ensures that all necessary concepts and relationships are included. While absolute completeness is not achievable, the MAD-Onto ontology aims to be as comprehensive as possible within its scope, incorporating insights from seminal works to develop a fine-grained ontology that fully conceptualizes mobile app development.
3. Conciseness: A concise ontology contains the minimum number of concepts and relationships necessary to represent the domain it models, avoiding redundancy. A concise ontology is easier to understand and use. For the MAD-Onto ontology, conciseness was achieved by carefully crafting the ontology to provide essential and non-redundant information about mobile app development. This focus on conciseness ensures that users can easily find the information they need and understand how the ontology functions.
4. Expandability: Expandability refers to the ability of an ontology to be easily modified to add new concepts and relationships. This is crucial because the domain it models is often dynamic and evolving. The MAD-Onto ontology is designed to be extensible and interoperable, allowing for easy modification by adding, removing, or altering axioms. It aligns with the four extensibility principles: ontology term reuse, semantic alignment, ontology design patterns (ODP) usage for new term generation and existing term editing, and community extensibility (He et al., 2018). This design ensures that the ontology can adapt to new knowledge and emerging use cases.
5. Sensitiveness: An ontology is considered sensitive if changes to the ontology significantly impact its core structure. The MAD-Onto ontology, while flexible and open to amendments, is carefully structured to ensure that essential core elements remain stable even when updates or changes are made. This balance between flexibility and stability ensures that the ontology can accommodate new information and modifications without compromising its foundational integrity.

The evaluation of the MAD-Onto ontology using these five criteria ensures a robust, reliable, and comprehensive framework for modeling mobile app development. By focusing on consistency, completeness, conciseness, expandability, and sensitiveness, the ontology is designed to be a valuable resource for developers, researchers, and other stakeholders in the mobile app development domain. This structured approach to ontology evaluation guarantees that MAD-Onto is both practical and adaptable, meeting current needs while being prepared for future advancements.

### 5.2 Evaluation at the ontology level

The evaluation at the ontology level is a systematic process to determine the robustness of an ontology. It examines the organization (structure), the significance (semantics), and the user-friendliness (usability) of the ontology. The structure pertains to the ontology’s arrangement, semantics to the definitions and connections within, and usability to the ease with which users can interact with the ontology. Several methodologies exist for this evaluation (Srinivasulu et al., 2014; Ajami and Mcheick, 2018). The equations of these methodologies are formulated in Equations 1–5 and discussed as follows:

- Vocabulary Size (VS): This metric quantifies the breadth of an ontology’s lexicon, encompassing classes, instances, and attributes. For MAD-onto, the VS is calculated as:

(1) VS=|C|+|I|+|P|

Where $\left(\left|C\right|\right)$ represents classes, $\left(\left|I\right|\right)$ instances, and $\left(\left|P\right|\right)$ properties. A substantial VS suggests a rich and intricate ontology, though it may also imply increased complexity in usage and maintenance. VS for MAD-onto: VS=85+162+23=270 . The Vocabulary Size of 270 indicates that MAD-onto has a moderate number of terms, which suggests it is neither too simple nor excessively complex. This size is likely to offer a comprehensive coverage of the domain without overwhelming the users with too many terms.

- Connectivity Ratio (CR): The CR assesses the ontology’s interconnectedness by comparing the number of links (edges) to concepts (nodes):

(2) CR=E/N

Where (E) is edges and (N) is nodes. A higher CR indicates a dense network of relationships, which could enhance or complicate comprehension, depending on the context. As for MAD-onto, $CR=\frac{117}{277}\approx 0.422$ . A Connectivity Ratio of approximately 0.422 suggests that, on average, each node (class, individual, or property) in MAD-onto is connected to less than one other node. This indicates that the ontology is not overly dense, which can be beneficial for users to understand and navigate the ontology without confusion from too many interconnections.

- Tree impurity (TIP): TIP metric quantifies the degree to which the inheritance hierarchy of an ontology deviates from a tree. It is a logical indication of how effectively inheritance connections are arranged in an ontology.

(3) $$\mathrm{TIP}=\frac{\left(E-N+1\right)}{N-1}$$

Where p is the proportion of child concepts in a specific category. A lower TIP value signifies a well-defined hierarchy, whereas a higher value points to potential ambiguities. TIP for MAD-onto is 0.321, this indicates that the MAD-onto ontology has a moderate level of hierarchy clarity. The majority of the concepts are well-defined and the hierarchy is relatively clear. This level of TIP is generally acceptable for ontologies, as it allows for some flexibility in categorization without causing too much confusion or uncertainty for users navigating the ontology.

- The entropy of ontology graph (EOG): is a metric that quantifies the complexity and information content of an ontology’s graph structure. It is rooted in the principle of information entropy, a concept that gauges the unpredictability or randomness present in a data set. The EOG metric evaluates the level of uncertainty or randomness within the structure of an ontology’s graph. It does this by considering both the quantity of nodes and edges in the graph, as well as the distribution of links between them. The EOG metric can be calculated using the following formula:

(4) $$EOG=-\overset{n}{\underset{x=1}{\sum }}p\left(x\right)\log_{2}\left(p\left(x\right)\right)$$

Where $p\left(x\right)$ denotes the likelihood of a specific connection type in the graph, such as the chance that two nodes are connected by a specific type of edge. The EOG metric ranges from 0 to $\log_{2}\left(N\right)\text{,}$ where N is the number of nodes in the ontology. $p\left(x\right)$ is computed by dividing the vertex’s degree, or the number of attributes associated with that class, by the total sum of all degrees of V for each node x in the graph. In particular, $p\left(x_{i}\right)$ can be computer as:


(5) $$p\left(x_{i}\right)=\frac{\deg\left(x_{i}\right)}{\sum_{v\in V}\deg\left(x\right)}$$

A higher EOG number suggests more uncertainty or unpredictability in the network structure, whereas a lower EOG value indicates a more organised and predictable graph.

The value of EOG for MAD-onto is almost one, demonstrating that the class structure of MAD-onto is adequate and reasonable.

### 5.3 Class-level evaluation: the ODQM approach

Brewster et al. (2004) introduced a method called “Ontology Design Quality Measure” (ODQM) to assess the quality and complexity of individual classes within an ontology. It analyzes various aspects of a class’s design through specific metrics.

- Number of Classes (NOC): This metric simply counts the total number of classes in the ontology. A higher NOC indicates a broader and deeper coverage of concepts within the domain. MAD-Onto’s NOC of 85 suggests a reasonable starting point, with the expectation of further expansion as the ontology matures.
- Number of Properties (NOP): This metric reflects the richness and complexity of relationships and attributes associated with classes. MAD-Onto’s NOP of 111 signifies a solid foundation for reasoning within the ontology.
- Number of Root Classes (NORC): This metric counts the classes without any parent classes (superclasses)—essentially the top level of the ontology hierarchy. MAD-Onto’s NORC of 15 indicates a well-defined structure with distinct but related core concepts.
- Relationship Richness (RR): This metric considers the count of non-inheritance relationships (like disjoint classes, equivalent classes, and object properties) to the count of subclass (inheritance) relationships. The higher the RR value, the more diverse the ontology is in terms of the types of relationships it includes. Conversely, a lower RR value would suggest an ontology that relies more heavily on inheritance relationships, which might be less expressive. MAD-Onto’s RR of 0.57 signifies a good level of richness in factual relationships based on its conceptual structure.

### 5.4 Evaluating using SWRL rules

The Semantic Web Rule Language (SWRL) is a powerful language designed for the Semantic Web to facilitate the formulation of rules and logical statements that encapsulate knowledge (Hassanpour et al., 2009; Horrocks et al., 2004). This language serves as a tool for representing complex knowledge structures that are not easily captured by the Web Ontology Language (OWL) alone. In the context of assessing ontologies, SWRL plays a crucial role. It allows for the creation of specific rules that can verify the internal consistency of an ontology and ascertain whether it adheres to predefined constraints. These rules are adept at articulating intricate constraints that surpass the capabilities of OWL. To conduct an ontology evaluation with SWRL, one must first define the necessary rules. Subsequently, these rules are implemented within the ontology through the use of a reasoning engine. This engine carefully examines the ontology to determine compliance with the rules, pinpointing any discrepancies or non-conformities in the process.

Employing SWRL rules in the evaluation phase is helpful in validating the ontology’s precision, comprehensiveness, and coherence. It ensures that the ontology not only aligns with the intended specifications and limitations but also upholds the quality standards expected of a robust knowledge representation system. Thus, SWRL enhances the reliability and utility of ontologies within the Semantic Web framework. By applying these rules, the MAD-Onto ontology can be evaluated and refined to ensure it is accurate, complete, and consistent with its intended requirements. We provide the following set of SWRL rules to evaluate the semantics of the developed ontology.

**Figure 6:**
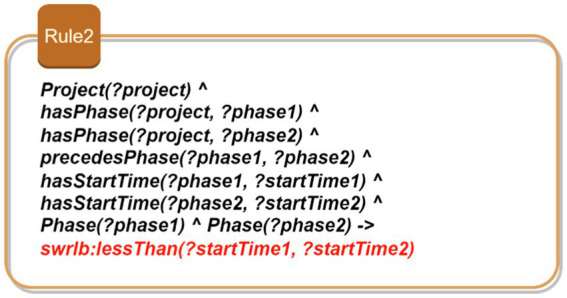
SWRL—Rule 2.

**Figure 7:**
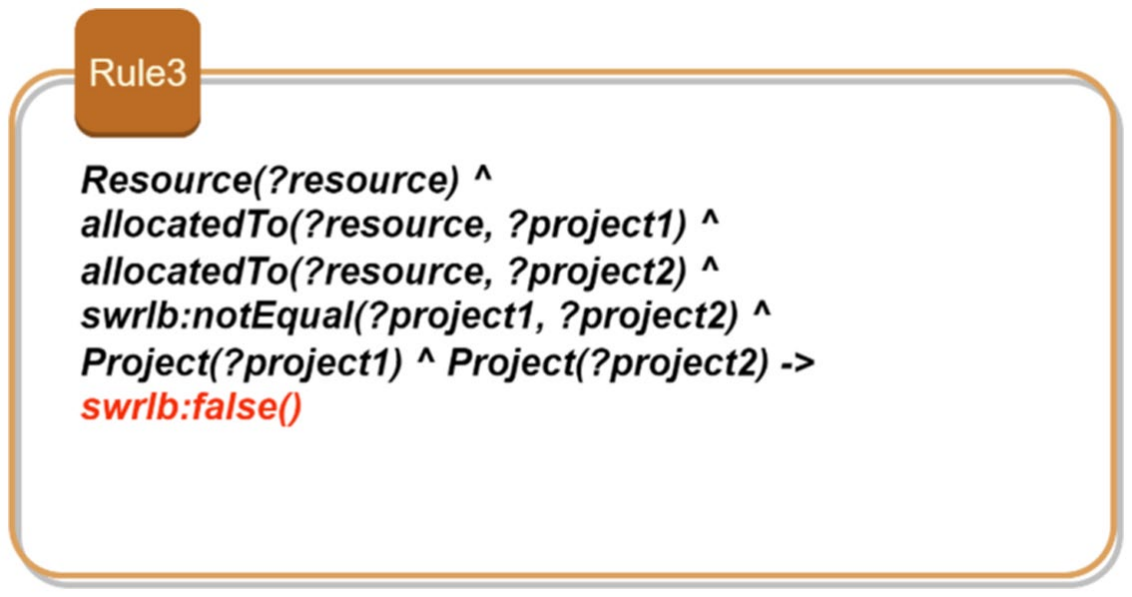
SWRL—Rule 3.

**Figure 8:**
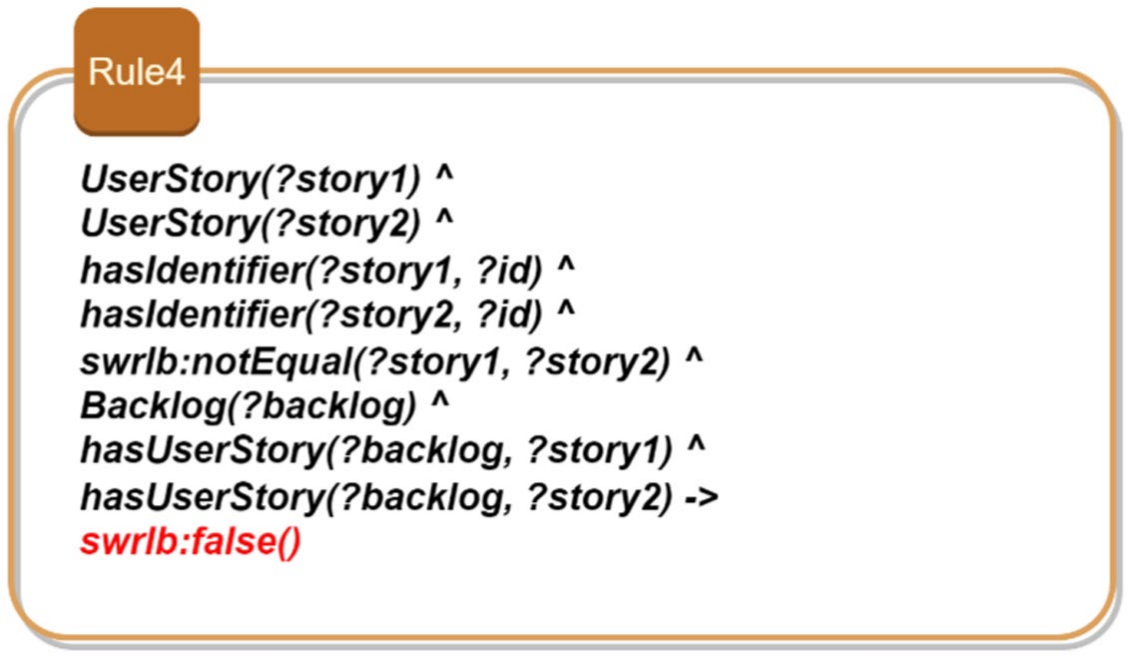
SWRL—Rule 4.

- Rule 1: Consistency of task assignments based on developer expertise: Figure 5 shows Rule 1 which aims to ensure that developers assigned to projects possess the required skills for each specific task. This rule ensures that only qualified developers are assigned to specific tasks within a project. The rule identifies a project and its associated tasks, each of which requires certain skills. It then checks whether the developers assigned to these tasks possess the required skills. By doing so, it maintains the consistency of task assignments, ensuring that each task is performed by a developer who is adequately qualified. This prevents unqualified personnel from taking on tasks they are not equipped to handle, thereby ensuring the quality and efficiency of the project development process.
- Rule 2: Verification of development phase order: As depicted in Figure 6, this rule enforces the correct sequence of development phases within a project. It identifies the phases associated with a project and ensures that they are completed in the order specified by the project plan. For instance, a “Design Phase” must precede the “Implementation Phase.” By verifying that the start time of each phase follows the chronological order, this rule ensures a logical and methodical progression of the project, preventing phases from being started out of order which could lead to project delays and inefficiencies.
- Rule 3: Prevention of resource allocation conflicts: This rule aims to prevent the same resource from being allocated to multiple projects simultaneously. As illustrated in Figure 7, this rule ensures that a resource, such as a developer or a testing device, is not assigned to more than one project at the same time. By identifying resources and their allocations, the rule checks for conflicts where a single resource is allocated to multiple projects concurrently. It prevents scheduling conflicts and overcommitment, ensuring that each resource is dedicated to a single project at any given time. This promotes efficient resource utilization and prevents bottlenecks that could arise from resource contention.
- Rule 4: Uniqueness of user stories: This rule enforces the uniqueness of user story identifiers within the project backlog. It identifies user stories and their respective identifiers, ensuring that no two user stories share the same identifier. By doing so, the rule prevents confusion and ambiguity, allowing each user story to be uniquely referenced and tracked. This is crucial for maintaining an organized and manageable backlog, where each user story can be individually identified and monitored for progress. Figure 8 demonstrates this rule.

Applying these SWRL rules to the MAD-Onto ontology significantly enhances the evaluation process by ensuring consistency, completeness, and adherence to best practices within the mobile app development domain. The rules help maintain a high standard of quality by verifying that developers are appropriately skilled for their tasks, phases are completed in the correct order, resources are efficiently utilized without conflicts, and user stories are uniquely identifiable. These checks are vital for maintaining the integrity and effectiveness of the ontology, ensuring that it accurately represents the knowledge and constraints of the mobile app development process. By using SWRL rules, we can systematically detect and address inconsistencies, redundancies, and other issues that might compromise the ontology’s utility and reliability, ultimately leading to more robust and reliable project outcomes.

### 5.5 Application of MAD-onto

To demonstrate the practical utility and effectiveness of MAD-onto, this section explores two comprehensive case studies that exemplify how the ontology addresses real-world challenges in MAD. These case studies illustrate MAD-onto’s ability to facilitate decision-making, enhance resource allocation, and optimise workflows in scenarios involving complex requirements and diverse technologies.

#### 5.5.1 Case Study 1: Cross-platform e-commerce app development with emerging technologies

In this case, an e-commerce company seeks to develop a cross-platform mobile app featuring advanced functionalities, including AR for virtual product try-ons, AI for personalized recommendations, and blockchain for secure payment processing. The project poses significant challenges due to the need for integrating diverse technologies, selecting appropriate development tools, and ensuring efficient task sequencing across a multidisciplinary team. MAD-onto effectively addresses these challenges by providing a structured framework for selecting the best-fit technologies and tools based on the project’s requirements. For instance, using MAD-onto’s EmergingTechnology and DevelopmentApproach classes, the project team identifies Unity 3D and ARKit as suitable frameworks for integrating AR capabilities on iOS devices, TensorFlow Lite for on-device AI-powered recommendations, and Hyperledger Fabric as the blockchain platform for secure payment transactions. These recommendations streamline the decision-making process, allowing the team to focus on implementation rather than exhaustive technology evaluations.

In addition to technology selection, MAD-onto enforces logical task sequencing through its integration with SWRL rules. For example, rules embedded in the ontology ensure that AR model rendering tasks cannot commence until the UI design phase is complete and that blockchain validation processes must precede app deployment. These rules are implemented using reasoning engines such as HermiT in Protégé, automatically flagging any deviations from the prescribed workflow and reducing the risk of project delays. Furthermore, MAD-onto facilitates optimal resource allocation by aligning team members’ expertise with specific tasks. The ontology’s Person and Task classes map developer roles to their required skills, ensuring that tasks such as blockchain integration and AR functionality are assigned to specialists in these domains. This structured allocation reduces inefficiencies and enhances team productivity. MAD-onto not only supports the technical and managerial aspects of this e-commerce app development project but also ensures that all phases are executed cohesively and efficiently, demonstrating its ability to navigate the complexities of multi-technology app development.

#### 5.5.2 Case Study 2: IoT-based health monitoring app with multi-device support

In this case, a healthcare provider aims to create an IoT-enabled mobile app that connects with wearable devices such as fitness trackers and smartwatches to monitor users’ health metrics in real-time. The app must ensure interoperability with a variety of devices, securely manage sensitive health data, and provide an intuitive user interface to enhance usability. The development team faces challenges in integrating IoT devices, addressing data privacy concerns, and balancing real-time functionality with offline capabilities. MAD-onto offers significant value in ensuring device interoperability through its DeviceAccessManager and MobilePlatform classes. By querying the ontology, the development team identifies Bluetooth Low Energy (BLE) as the optimal protocol for establishing communication between the app and IoT devices. Additionally, libraries such as RxAndroidBLE and CoreBluetooth are recommended for seamless integration with Android and iOS platforms, respectively. This structured guidance accelerates the implementation process and ensures compatibility with a wide range of devices.

To address data security and privacy concerns, MAD-onto’s SecurityFeature and BackendDevelopment classes provide best practices for securely managing health data. The ontology recommends employing AES-256 encryption for end-to-end data protection and highlights compliance with regulations such as HIPAA and GDPR. By incorporating these recommendations, the app ensures that users’ health information is handled securely and adheres to global privacy standards.

MAD-onto also supports offline functionality, a critical requirement for users in areas with limited internet connectivity. Through the ExecutionEnvironment and MemoryManagement classes, the ontology suggests caching data locally using SQLite, enabling users to access key features like viewing health insights and historical metrics without a network connection. This capability significantly enhances user experience and broadens the app’s accessibility. In addition to these technical aspects, MAD-onto aids in optimizing team workflows. The ontology’s TestingStrategy class supports the creation of rigorous test cases for validating the app’s performance under varying network conditions and ensuring its compatibility across devices. By incorporating these tests, the development team can confidently deliver a robust and user-friendly health monitoring solution. This case study illustrates how MAD-onto facilitates IoT-enabled app development by addressing the unique challenges of interoperability, security, and usability, demonstrating its ability to adapt to the evolving demands of the healthcare domain.

These case studies highlight MAD-onto’s versatility and its capacity to address complex and diverse challenges in MAD. Whether integrating cutting-edge technologies for e-commerce or ensuring secure and seamless IoT connectivity for healthcare, MAD-onto provides a structured, standardized approach to managing intricate workflows and technical requirements. Its ability to guide developers in selecting tools, sequencing tasks, and allocating resources effectively makes it an indispensable resource for modern app development. These examples not only demonstrate the ontology’s theoretical robustness but also validate its practical utility in addressing real-world application needs.

## 6 Conclusion and future work

The field of MAD has swiftly become a vital component of contemporary technology, spurring innovation across numerous industries and reshaping user experiences worldwide. Due to the ever-changing nature of mobile technology, developers must adeptly maneuver through a multifaceted environment of platforms, devices, and user demands. Efficient knowledge management and sharing are critical to overcoming these challenges, facilitating streamlined development processes and improved collaboration among stakeholders. In this context, ontologies have proven to be invaluable tools for organizing and standardizing domain-specific knowledge. MAD-onto (Mobile App Development Ontology) is a comprehensive framework designed to address the complexities and challenges inherent in mobile app development. By structuring and standardizing domain-specific knowledge, MAD-onto facilitates efficient knowledge management, sharing, and collaboration among stakeholders. This ontology captures essential concepts, attributes, and relationships within the mobile app development domain, enabling developers to navigate the intricate landscape of platforms, devices, and user requirements effectively. By leveraging MAD-onto, developers can ensure that all relevant aspects of mobile app development are considered, leading to more streamlined processes and improved project outcomes.

The ontology is organized hierarchically, beginning with general classes and branching into more specific subclasses, which encompass various facets of mobile app development, such as development approaches, tools, methodologies, and best practices. MAD-onto also defines properties and relationships between these classes, providing a detailed and interconnected view of the domain. The use of MAD-onto enhances consistency, completeness, conciseness, expandability, and sensitivity in the development process. By providing a structured framework, MAD-onto not only aids in addressing current development challenges but also supports the continuous evolution and extension of knowledge as new technologies and practices emerge in the field.

## Data Availability

The raw data supporting the conclusions of this article will be made available by the authors, without undue reservation.
